# The transcription factor BACH1 at the crossroads of cancer biology: From epithelial–mesenchymal transition to ferroptosis

**DOI:** 10.1016/j.jbc.2021.101032

**Published:** 2021-07-30

**Authors:** Kazuhiko Igarashi, Hironari Nishizawa, Yuriko Saiki, Mitsuyo Matsumoto

**Affiliations:** 1 Department of Biochemistry, Tohoku University Graduate School of Medicine, Sendai, Japan; 2 Center for Regulatory Epigenome and Diseases, Tohoku University Graduate School of Medicine, Sendai, Japan; 3 Department of Investigative Pathology, Tohoku University Graduate School of Medicine, Sendai, Japan

**Keywords:** BACH1, NF-E2-related factor 2, transcription factor, cancer, metastasis, epithelial–mesenchymal transition, iron, heme, ferroptosis, glycolysis, AP-1, activator protein-1, BACH1, BTB and CNC homology 1, BTB, broad-complex, tramtrack, bric-a-brac, bZip, basic leucine zipper, CNC, cap‘n’collar, EMT, epithelial-to-mesenchymal transition, ES, embryonic stem, GRN, gene regulatory network, HDAC1, histone deacetylase 1, HMMR, hyaluronan-mediated motility receptor, HO-1, heme oxygenase-1, MARE, MAF recognition element, MET, mesenchymal-to-epithelial transition, PDAC, pancreatic ductal adenocarcinoma, ROS, reactive oxygen species

## Abstract

The progression of cancer involves not only the gradual evolution of cells by mutations in DNA but also alterations in the gene expression induced by those mutations and input from the surrounding microenvironment. Such alterations contribute to cancer cells' abilities to reprogram metabolic pathways and undergo epithelial-to-mesenchymal transition (EMT), which facilitate the survival of cancer cells and their metastasis to other organs. Recently, BTB and CNC homology 1 (BACH1), a heme-regulated transcription factor that represses genes involved in iron and heme metabolism in normal cells, was shown to shape the metabolism and metastatic potential of cancer cells. The growing list of BACH1 target genes in cancer cells reveals that BACH1 promotes metastasis by regulating various sets of genes beyond iron metabolism. BACH1 represses the expression of genes that mediate cell–cell adhesion and oxidative phosphorylation but activates the expression of genes required for glycolysis, cell motility, and matrix protein degradation. Furthermore, BACH1 represses *FOXA1* gene encoding an activator of epithelial genes and activates *SNAI2* encoding a repressor of epithelial genes, forming a feedforward loop of EMT. By synthesizing these observations, we propose a “two-faced BACH1 model”, which accounts for the dynamic switching between metastasis and stress resistance along with cancer progression. We discuss here the possibility that BACH1-mediated promotion of cancer also brings increased sensitivity to iron-dependent cell death (ferroptosis) through crosstalk of BACH1 target genes, imposing programmed vulnerability upon cancer cells. We also discuss the future directions of this field, including the dynamics and plasticity of EMT.

Cancer cells are suggested to reflect an evolutionary process in which genetic mutations modify cells, giving rise to variations. The ensuing struggle for existence (1) of these cells within the body selects for improved and/or adapted forms of cancer cells in terms of so-called cancer hallmarks (2) including proliferation, invasion, and metastasis, as well as stress resistance. Some cancer cells eventually dominate not only competing fellow cancer cells but also the surrounding normal cells. During the struggle for existence, cancer cells alter their properties and functions by not only genetic mutations but also changes in their gene expression in response to their microenvironment and therapeutic interventions (3). The modulability of gene expression, including epigenetic alterations, may be another foundation of cancer cell evolution, as heterogeneity thus formed allows for the selection of better-fitted cells among genetically similar cells. Therefore, some of the sinister properties of cancer cells such as metastasis, which underlie the difficulties in treating patients with cancer, may not be entirely attributable to genetic mutations alone.

Metastasis depends on alterations in the gene expression and involves multiple steps of local invasion, intravasation, extravasation, and reproliferation (4). Epithelial-to-mesenchymal transition (EMT) can explain many of the alterations incurred by cancer cells during metastasis, which reduces the cell adhesion capability and increases mobility and invasiveness (5). Transcription factors known to regulate EMT include ZEB1, ZEB2, SNAI1 (SNAIL), SNAI2 (SLUG), and TWIST1 and have been suggested to promote cancer progression, including metastasis (5, 6, 7, 8). However, the direct target genes of these EMT-related transcription factors, shared among them or unique to one of them, largely remain elusive (8).

Transcription factors play critical roles in the survival of cancer cells, which are placed under stress derived from their hostile microenvironments of low oxygen, low nutrients, including glucose, and attack by immune cells. Changes in the signaling and metabolism within cancer cells impose endogenous stress. The survival of these cells despite such stress is dependent on their altered gene expression, choreographed by transcription factors. Hypoxia-responsive factor (HIF1A) induces neoangiogenesis (9, 10) and reprogramming of glucose metabolism from oxidative phosphorylation to the combination of glycolysis and oxidative phosphorylation (aerobic glycolysis) (9, 11). Heat shock factor HSF1 maintains protein homeostasis (12). Activator protein-1 (AP-1) transcription factors, such as JUN and FOS, which are characterized by the presence of basic leucine zipper (bZip) DNA-binding domain, are the downstream effector molecules of the mitogen-activated protein kinase and extracellular signal-regulated kinase signaling cascades and promote stress resistance against inflammation and oxidative stress (13). NF-E2-related factor 2 (NFE2L2), another transcription factor with a bZip domain, supports tumor cell proliferation in part by activating oxidative stress response (14, 15, 16).

The focus of this review is BTB and CNC homology 1 (BACH1) (17, 18), which regulates iron- and heme-related genes in normal cells (19, 20). Investigations on various types of human cancers have established that BACH1 promotes cancer progression *via* multiple mechanisms (Fig. 1, *A* and *B*). We first review the basic structure, function, and regulation of this transcription factor. We then summarize recent findings on the function of BACH1 in cancer cells and examine how the properties of cancer cells, including EMT, altered metabolism, metastasis, angiogenesis, and epigenetics, can be explained based on the established and/or putative BACH1 target genes. We propose a “two-faced BACH1 model” to integrate diverse functions of BACH1 in cancer cells. We additionally consider how other features of cancer cells, such as their dynamics and plasticity, and ferroptosis (iron-dependent cell death) may be shaped by BACH1. These findings make a compelling case that examining the roles of transcription factors is key to the global understanding of cancer biology.

**Figure 1:**
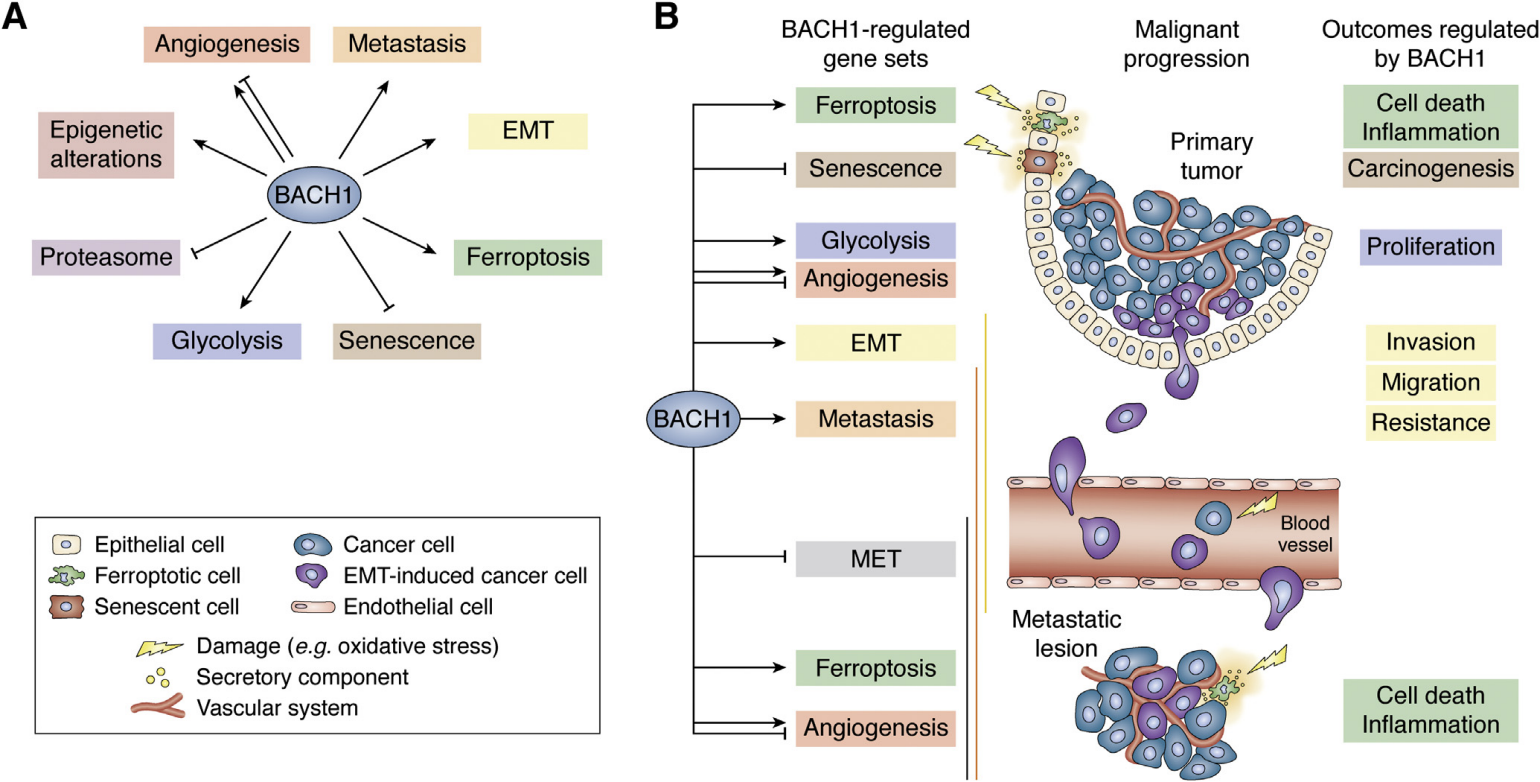
**BACH1 promotes metastasis by multiple mechanisms.***A*, BACH1 regulates multiple biological steps to promote cancer progression. The effects of BACH1 onto indicated events are shown with an *arrowhead* (promotion) or *stop line* (inhibition). *B*, malignant progression of cancer is depicted along the center line, with types of cells, involved in these steps, depicted at the *left of the panel* (below *panel A*). Shown on the left are gene categories that are related to cancer progression and are regulated by BACH1. The net effects of BACH1 upon those categories are indicated with an *arrowhead* (promotion) or *stop line* (inhibition). On the right are outcomes of the regulation by BACH1 in normal or malignant cells. The *yellow*, *orange*, and *gray vertical lines* indicate spatial extent of EMT, metastasis, and MET, respectively. BACH1, broad-complex, tramtrack, bric-a-brac and cap‘n’collar homology 1; EMT, epithelial-to-mesenchymal transition; MET, mesenchymal-to-epithelial transition.

## Fundamental properties and function of BACH1

### Molecular evolution and structure

**Figure 2:**
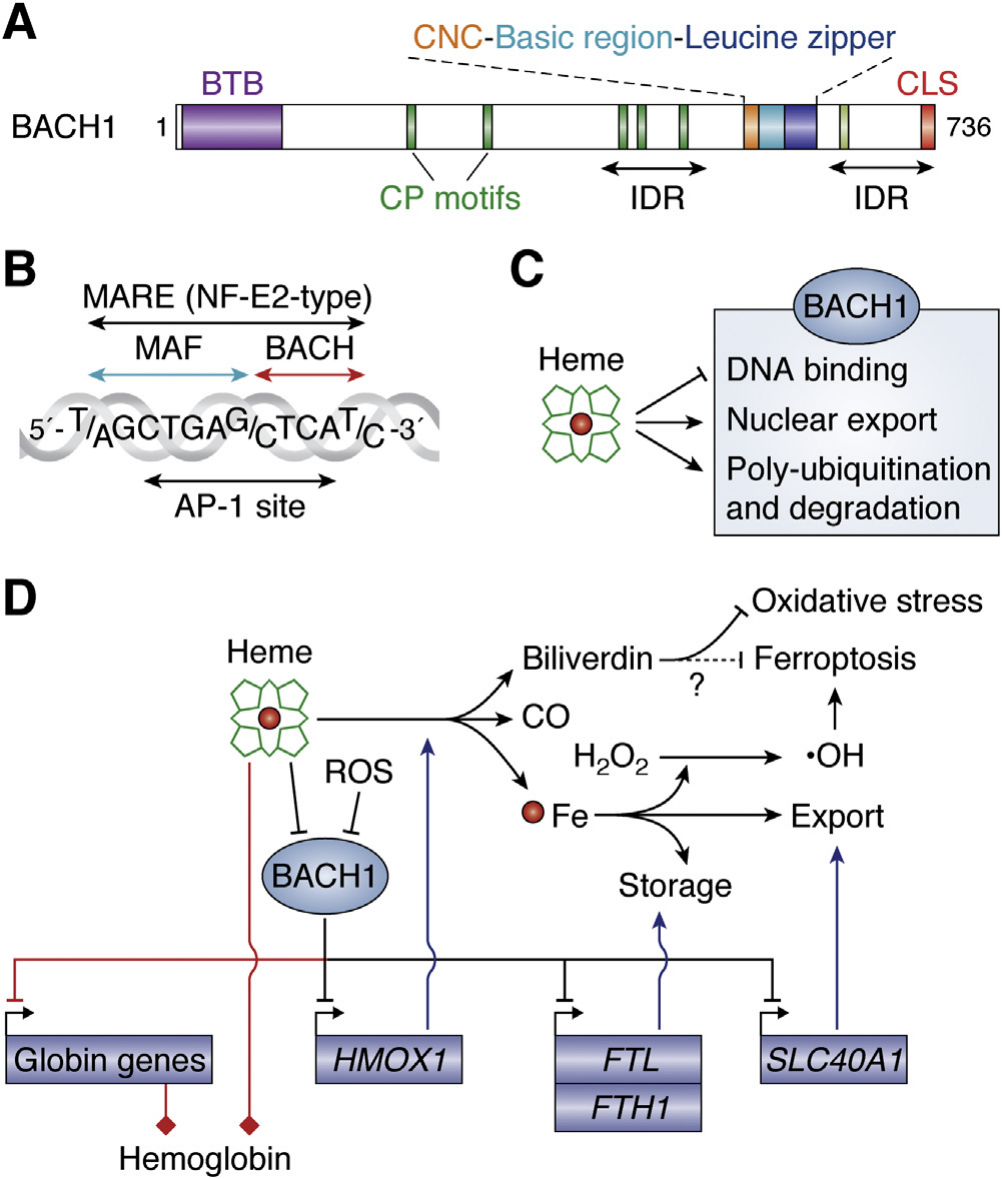
**Structure and function of BACH1.***A*, schematic representation of BACH1. Domains are shown in *colored boxes*. *Green boxes* indicate CP motifs. *B*, BACH1-binding DNA sequence MARE (NF-E2-type) and its relationship with AP-1-binding sequence (5′-TGAG/CTCA-3′). MAF and BACH1 recognize the longer and shorter halves of the motif, respectively. *C*, effects of heme on BACH1. *D*, gene regulatory network (GRN) of heme-BACH1-target genes. *Red lines* indicate regulatory interactions in erythroid cells. *Red* and *black lines* indicate regulation of the target genes by BACH1 in erythroid and nonerythroid cells, respectively, whereas *blue lines* depict functions of the products of BACH1 target genes. Generation of hemoglobin using globin and heme is indicated with the *lines with squares*. It is not established yet whether biliverdin suppresses ferroptosis or not. AP-1, activator protein-1; BACH1, BTB and CNC homology 1; BTB, broad-complex, tramtrack, bric-a-brac domain; CLS, cytoplasmic localization signal; CNC, cap‘n’collar region; CP, cysteine–proline; IDR, intrinsically disordered region; MARE, MAF recognition element.

Among the human transcription factors, there are 61 transcription factors with a bZip DNA-binding domain, thus constituting the fourth most abundant family of transcription factors after the zinc finger, homeodomain, and basic helix–loop–helix protein families (21). The bZip family can be further classified into subfamilies according to their structural features. BACH1 and BACH2 are the only bZip factors with the combination of broad-complex, tramtrack, bric-a-brac (BTB) and bZip domains (17) (Fig. 2*A*). However, their bZip domains are closely related to those of the cap‘n’collar (CNC) subfamily, which is characterized by the presence of a short, evolutionarily conserved segment preceding the bZip domain. This segment was originally found in *Drosophila* transcription factor CNC (22), from which the etymology of the subfamily name is derived. It is required for DNA binding (23) and is shared among BACH1, BACH2, NFE2 (NF-E2 p45), NFE2L1 (NRF1), NFE2L2 (NF-E2-related factor 2), which regulates oxidative stress response, and NFE2L3 (NRF3) (24). The *NFE2*, *NFE2L1*, *NFE2L2*, and *NFE2L3* genes are each linked to one or the other of the four clusters of *HOX* genes, which encode HOX family transcription factors important for the body plan in development. Because quadruplication of *HOX* clusters reflects two consecutive genome duplications during the evolution of vertebrates from deuterostomes (25), *NFE2*, *NFE2L1*, *NFE2L2*, and *NFE2L3* may have adopted roles specific to vertebrate. In contrast, *BACH1* and *BACH2* are not linked to the *HOX* gene clusters, suggesting that the presumptive ancestor gene had first been separated into *BACH* and *NFE2* type genes before or during the evolution of chordates, which possess a single *Bach* homolog (26). These factors form heterodimers with the small MAF oncoproteins (MAFF, MAFG, and MAFK) to bind to a DNA element referred to originally as an MAF recognition element (MARE) (27) and recently as CNC–sMAF–binding element (28, 29) (Fig. 2*B*). Because MARE embeds a motif for activator protein-1, MARE and its related motifs can be bound by AP-1 factors such as JUN and FOS (30).

The BTB domain mediates homophilic protein interaction, and hence, transcription factors with the BTB domain may thus mediate long-range interaction of *cis* elements (31, 32). The BTB domain of the zinc finger protein BCL6 mediates interaction with corepressors as well (33). The BTB domain of BACH1 is also bound by ubiquitin E3 ligase adaptor proteins (34, 35), and the BACH1 BTB domain directly binds to NANOG (36).

BACH1 and BACH2 function as transcription repressors by recruiting corepressors NCOR1, NCOR2, and the histone deacetylases such as histone deacetylase 1 (HDAC1) and histone deacetylase 3 (37, 38, 39, 40). Because their binding sites embed the AP-1 site, these transcription factors may compete with AP-1 factors to repress their shared target genes (Fig. 2*B*) (30). Note that while BACH target genes may also be regulated by AP-1 factors, AP-1 target genes are not necessarily regulated by BACH1 or BACH2, as the recognized sequences for the heterodimers of BACH1 or BACH2 with the small MAF proteins are longer than those of AP-1 factors (Fig. 2*B*). The antagonistic relationship between BACH2 and AP-1 is well established in the regulation of T cell activation and regulatory T cell differentiation (41, 42, 43).

### Mutual regulation of BACH1 and heme

BACH1 and BACH2 are the only bZip factors known to be regulated by direct binding of heme, a prosthetic group involved in the transport of oxygen and electron (44, 45, 46, 47, 48, 49, 50). These factors, together with other heme binding proteins, suggest new roles of heme sensors and gas sensors (51). Conventional heme proteins, such as globin, myoglobin, and cytochrome c, bind to heme *via* well-structured protein regions (52). In contrast, heme binds to BACH1 and BACH2 *via* cysteine–proline motifs embedded in an intrinsically disordered region (44, 45, 46, 47, 48, 49, 50) (Fig. 2*A*). Heme inhibits their DNA-binding activity (44, 46, 53) and induces nuclear export (54, 55), polyubiquitination, and subsequent degradation (46, 56) (Fig. 2*C*).

BACH1 represses target genes involved in the utilization or mobilization of iron, including *HMOX1* for heme oxygenase-1 (HO-1) (57), *FTH1* and *FTL* for ferritin heavy- and light-chain subunits (58), and *SLC40A1* for ferroportin (59) (Fig. 2*D*). Heme induces the expression of these genes by inactivating BACH1. In erythroid cells, when iron and thus heme synthesis are sufficient, BACH1 is inactivated by heme, leading to the expression of globin genes and abundant synthesis of hemoglobin (20, 60). Under iron-deficient conditions, BACH1 resumes its activity, leading to a reduction in the globin gene expression and thus a rebalanced synthesis of globin protein and heme (20) (Fig. 2*D*). This is important for ensuring the tolerance of iron deficiency, possibly by avoiding toxicity of free globin proteins. Regulation of the homeostasis of iron and related reactions, such as electron or oxygen metabolism, may be the archetypal function of BACH1 and BACH2.

### Regulation of oxidative stress response and senescence by BACH1

Various enzymatic and nonenzymatic reactions within cells generate reactive oxygen species (ROS). Alterations in ROS metabolism and their pathological meaning in cancer development have recently been extensively reviewed (61, 62). While ROS are counteracted by antioxidants generated in cells or taken up as nutrients, excess ROS interact with and damage molecules such as DNA, RNA, proteins, and lipids, resulting in oxidative stress. ROS inactivate BACH1 by oxidizing its cysteine residue or by promoting its phosphorylation by a tyrosine kinase (63, 64), leading to the expression of its target genes, including *Hmox1*, whose product is a potent antioxidant enzyme (19) (Fig. 2*D*). Therefore, BACH1 is regulated by ROS, tuning the homeostasis of ROS in part through the *Hmox1* regulation.

Along with the repression of oxidative stress–responsive genes, BACH1 inhibits oxidative stress–induced cellular senescence (38). Normal cells undergo cellular senescence, a state lacking proliferation activity (65, 66, 67), in response to various stress stimuli, including oxidative stress and oncogene stress (68). Cellular senescence is one of the defense mechanisms for preventing cancer (68, 69, 70) and is driven by the tumor suppressor p53 (68, 71, 72). BACH1 suppresses cellular senescence of mouse embryonic fibroblasts by inhibiting the function of p53. It recruits HDAC1 to some of the p53 target genes that are required for senescence, thereby repressing their expression (38, 73).

Taken together, these data paint a clear picture of the many roles played by BACH1. We will next explore BACH1's links to cancer metastasis, which will help demonstrate how these roles intersect with cancer biology.

## Functions of BACH1 in cancer metastasis

Experiments using mouse xenograft models of human cancer cells, with genetic manipulations of BACH1, have established that BACH1 promotes metastasis of breast cancer (74, 75, 76), non–small cell lung cancer (77, 78), pancreatic ductal adenocarcinoma (PDAC) (79), and ovarian cancer (80). Although these models provide compelling evidence supporting the importance of BACH1, additional genetic models such as oncogene-driven cancer development in mice need to be tested. The results of *in vitro* assays also support the involvement of BACH1 in the metastatic properties of cancer cells. Invasion and migration activities of PDAC cells, esophageal cancer cells, colon cancer cells, and ovarian cancer cells *in vitro* are reduced upon BACH1 knockdown (79, 80, 81, 82, 83, 84). When overexpressed in colon or ovarian cancer cells, BACH1 enhanced tumor growth in transplanted mice, but metastasis was not investigated (81). An increased expression of BACH1 was shown to be correlated with a poorer prognosis of patients with breast, pancreatic, colorectal, ovarian, and esophageal cancers as well as glioblastoma (79, 80, 81, 84, 85, 86). Taken together, these observations indicate that cancer cells acquire metastatic potential when the activity of BACH1 is increased.

While many of the established target genes of BACH1 in normal cells are related to metabolism of iron, heme, and ROS (Fig. 2*D*), its target genes in cancer cells appear more diverse than those in normal cells (Table 1), without any apparent direct connection to iron or heme. The list includes genes whose direct regulation by BACH1 has been established by a combination of chromatin immunoprecipitation and knockdown of BACH1. We expect that more genes have yet to be discovered. Suggested target genes of BACH1 in the promotion of EMT and/or metastasis include those responsible for cell–cell adhesion of epithelial cells, transcription factors and other regulators for EMT, and enzymes for glycolysis, mitochondrial electron transport components, and matrix proteases (Table 1). Based on the list of its target genes in cancer cells, two types of models depicting the mechanism underlying BACH1-mediated metastasis can be envisioned: silencing of epithelial properties, including cell–cell adhesion, resulting in the promotion of EMT by BACH1, and the modulation of cancer cell metabolism by BACH1 (Fig. 1*B*).

**Table 1:** Direct BACH1 target genes in cancer cells

Cancers	Gene	Function	Effect	Evidence	Reference
Breast	*COX15*	Cytochrome c oxidase assembly	Repression	ChIP, RT-PCR	Lee *et al.* (76)
Breast	*UQCRC1*	Cytochrome b-c1 complex subunit	Repression	ChIP, RT-PCR	Lee *et al.* (76)
Breast	*ATP5D*	ATP synthase subunit delta, mitochondrial	Repression	ChIP, RT-PCR	Lee *et al.* (76)
Breast	*PDK1*	Phosphorylation of the pyruvate dehydrogenase subunits	Activation	ChIP, Western	Lee *et al.* (76)
Breast	*MMP1*	Collagenase	Activation	ChIP, Western, reporter assay	Yun *et al.* (74) Liang *et al.* (75)
Breast	*CXCR4*	Chemokine receptor	Activation	ChIP, Western, reporter assay	Yun *et al.* (74)
Breast	*PEBP1* (*RKIP*)	Modulation of kinase signaling	Repression	ChIP, reporter assay	Lee *et al.* (196)
Breast	*ROCK1*	Downstream effector of Rho	Activation	ChIP, RT-PCR	Yesilkanal *et al.* (100)
Breast	*BACH1*	Gene regulation	Repression	ChIP, reporter assay	Lee *et al.* (196)
Breast	*IL11*	Inflammation	Activation	ChIP (MAFF), RT-PCR	Moon *et al.* (101)
Colorectal	*BACH1*	Gene regulation	?	ChIP-Seq	Ying *et al.* (186)
Lung	*HK2*	Glycolysis	Repression	ChIP-Seq, RNA-Seq	Wiel *et al.* (78)
Lung	*GAPDH*	Glycolysis	Repression	ChIP-Seq, RNA-Seq	Wiel *et al.* (78)
Pancreas	*HMOX1*	Heme degradation	Repression	ChIP, Western	Huang *et al.* (112)
				ChIP-Seq	Sato *et* *al.* (79)
Pancreas	*FOXA1*	Epithelial gene expression	Repression	ChIP-Seq, RT-PCR	Sato *et al.* (79)
Pancreas	*CLDN3*	Tight junction	Repression	ChIP-Seq, RT-PCR	Sato *et al.* (79)
Pancreas	*CLDN4*	Tight junction	Repression	ChIP-Seq, RT-PCR	Sato *et al.* (79)
Pancreas	*RKP2*	Desmosome	Repression	ChIP-Seq, RT-PCR	Sato *et al.* (79)
Pancreas	*SNAI2*	EMT	Activation	ChIP-Seq, RT-PCR	Sato *et al.* (79)
Ovarian	*SNAI2*	EMT	Activation, depending on HMGA2	ChIP, Western	Han *et al.* (80)
Cholangiocarcinoma	*PMSA5*^a^	Proteasome	Repression	ChIP, RT-PCR	Jiang *et al.* (144)
Colorectal melanoma	*CDKN2A*^b^	Cell cycle	Repression	ChIP, RT-PCR	Fang *et al.* (126, 127)

Abbreviation: ChIP, chromatin immunoprecipitation.

a Other genes of proteasome components, including *PSMB1* and *PSMB2*, may also be regulated.

b Other genes in the category of CpG island methylator phenotype are also directly repressed by BACH1.

### Promotion of EMT by BACH1

**Figure 3:**
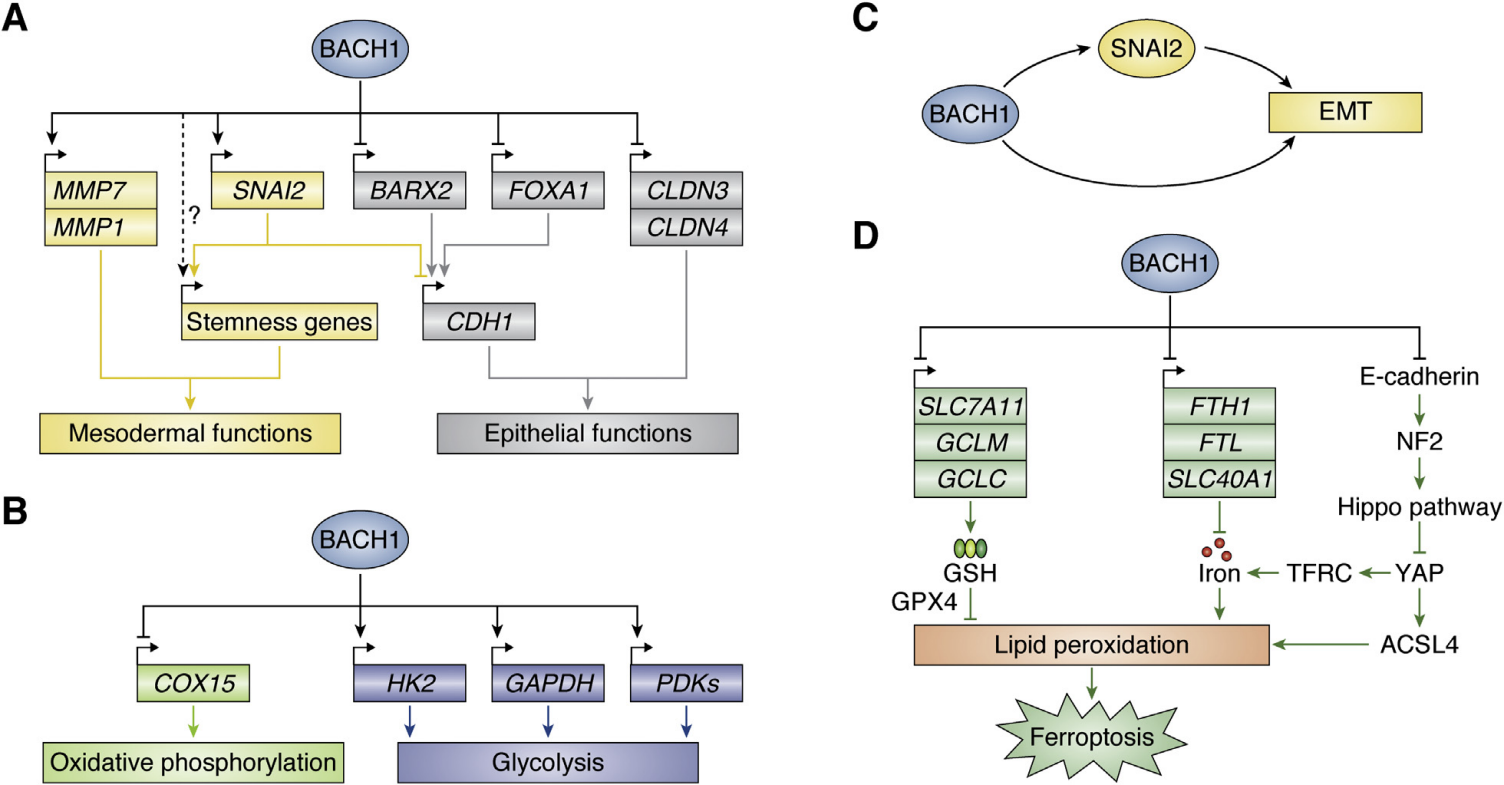
**BACH1 GRNs for cancer cell progression.***A*, EMT is regulated by BACH1 GRN that silences genes critical for epithelial cell structure and function. Some of the mesodermal genes are also directly activated by BACH1. Regulatory interactions mediated by gene products are depicted with *lines* between the genes or with phenotypic outcomes (*boxed*). A *line with an arrow* indicates activation, whereas a *blunt-end line* indicates repression. *B*, energy metabolism is regulated by BACH1 GRN that activates genes critical for glycolysis. Some of the genes for electron transfer chain are also directly repressed by BACH1. *C*, feedforward loop for EMT is generated by the repressor and activator activities of BACH1 together with *SNAI2*. *D*, BACH1 promotes ferroptosis in cancer cells by repressing genes central to the four pathways involved in the execution of ferroptosis (GSH-mediated protection, iron-mediated lipid peroxidation, and E-cadherin-NF2-Hippo-YAP pathways). The GRN is depicted as in Figure 2. BACH1, BTB and CNC homology 1; EMT, epithelial-to-mesenchymal transition; GRN, gene regulatory network.

BACH1 represses the expression of genes important for epithelial cell functions in PDAC cells. Specifically, BACH1 directly binds to a set of genes important for the epithelial structure to repress their expression in PDAC cells, including *CLDN3* and *CLDN4*, each encoding claudin protein, which is involved in the formation of the tight junction of epithelial cells (79) (Table 1). A reduction of these molecules is known to contribute to EMT (87). Aside from the genes involved in cell–cell adhesion, BACH1 regulates additional transcription factor genes that may be directly involved in the process of EMT, including *FOXA1* and *SNAI2*, forming a gene regulatory network (GRN), which is composed of transcription factors and their target genes, for EMT (Table 1, Fig. 3*A*). Depending on these transcription factors, BACH1 is connected indirectly to additional downstream genes for the epithelial structures as well. The expression of E-cadherin gene *CDH1* is low in cancer cells with a high *BACH1* expression, and a reduced expression of *CDH1* is dependent on *BACH1* (79, 80, 84). Because FOXA1 directly activates the expression of *CDH1* (88), FOXA1 may mediate the connection between *BACH1* and *CDH1* (Fig. 3*A*). While a reduced function of FOXA1 is involved in the progression and metastasis of cancers derived from digestive organs (88, 89, 90), some studies suggest its role in the promotion of metastasis in pancreatic, breast, and prostate cancers (91, 92, 93, 94). Therefore, the outcome of the BACH1–FOXA1 axis may be dependent on the cell context. *SNAI2* encodes an archetypal transcription factor driving EMT by repressing genes involved in cell–cell adhesion and promoting the stem cell function (95, 96, 97). *SNAI2* is directly activated by BACH1 in PDAC, ovarian cancer, and esophageal squamous cell carcinoma cells (79, 80, 84), suggesting that the BACH1–SNAI2 axis likely contributes to the silencing of epithelial genes.

In addition to reduced cell–cell adhesion, the degradation of matrix components and increased motility are required for cancer cell metastasis (98). BACH1 promotes the expression of matrix metallopeptidase: it directly activates the expression of *MMP1* in breast cancer cells (75) and possibly *MMP9* as well (99). BACH1 also directly enhances the expression of genes involved in cell motility and invasiveness, including *ROCK1* in breast cancer (100). Therefore, BACH1 promotes the invasiveness of cancer cells by enhancing their ability to degrade extracellular matrix and increasing their motility. The heterodimer of BACH1 and its partner MAFF induce the expression of IL-11 in breast cancer cells to promote invasiveness (101). BACH1 may further affect the destination of metastasizing cancer cells. CXCR4 is known to increase cell migration ability and promote cancer metastasis (102, 103). BACH1 has been reported to promote the expression of *CXCR4* in colorectal (81) and breast cancers (75, 99, 104). However, no published study has examined whether or not BACH1 directly regulates *CXCR4*.

The contribution of BACH1 to metastasis is further supported by several reports showing that genes whose expression is altered in EMT are enriched with DNA motifs of AP-1. Accessible chromatin regions occurring in the early stages of EMT are enriched with DNA motifs of AP-1 and MAF transcription factors (105). Another study (106) used multiple cancer cell lines and EMT-inducing factors to reveal a core transcriptional program of EMT. The genes in this category were enriched with those carrying AP-1 and BACH2 motifs. Considering the relationship of BACH1-binding and AP-1-binding sequences (Fig. 2*B*), these chromatin regions may also be regulated by BACH1.

### Promotion of aerobic glycolysis by BACH1

Direct target genes of BACH1 in breast cancer include many of those involved in the function of mitochondria, including oxidative phosphorylation. BACH1 represses oxidative phosphorylation in breast cancer cells (76), which is consistent with a glycolytic pattern of gene expression in metastatic triple-negative breast cancer cells (107). BACH1 expression shows an inverse correlation with the expression of genes related to the electron transport chain. BACH1 binds directly to some of these genes, including *COX15*, to repress their expression. Notably, mitochondrial respiration and the ATP contents are increased upon BACH1 knockdown (76). BACH1 promotes metastasis in non–small-cell lung cancer by promoting cell migration and invasion (77, 78, 108). The mechanism underlying these effects has been suggested to involve the promotion of glycolysis by BACH1 (78). BACH1 activates the expression of *PDK* genes, which encode the pyruvate dehydrogenase kinase required for the inactivation of pyruvate dehydrogenase, *HK2*, which encodes hexokinase in the first step of glycolysis, and *GAPDH*, which encodes glyceraldehyde-3-phosphate dehydrogenase in the glycolytic pathway (76, 78). BACH1 can restrict dependency on oxidative phosphorylation and promote aerobic glycolysis in cancer cells (Fig. 3*B*), a phenomenon known as the Warburg effect (2, 109). However, the regulation of genes related to glycolysis and oxidative phosphorylation may be specific to certain types of cancers. For example, *HK2* and *GAPDH* were not found to be bound and regulated by BACH1 in a previous study of PDAC cells (79). It will be important to investigate unique and overlapping target genes of BACH1 in cancer cells. The roles of BACH1 in reprogramming of cancer cell metabolism have been reviewed recently (110). How changes in metabolism lead to enhanced metastasis of cancer cells remains to be explored.

## Other roles of BACH1 in cancer progression

Additional mechanisms are also involved in the promotion of cancer progression by BACH1 (Fig. 1*A*). In addition, BACH1 possesses the ability to restrict cancer progression under some conditions. Therefore, its roles in cancer progression are multifaceted.

### Promotion of angiogenesis by BACH1

Angiogenesis is often a rate-limiting step of cancer proliferation (2). BACH1 in not only cancer cells but also surrounding stromal cells regulates angiogenesis. In one report, the overexpression of BACH1 in human ovarian cancer cells increased the expression of *VEGFC* and promoted both angiogenesis and lymphangiogenesis when transplanted in mice (111). Studies in zebrafish showed that Bach2 paralogs are essential for developmental angiogenesis (111). In contrast, another report showed that BACH1 inhibits angiogenesis by pancreatic cancer cells (112). Studies in other systems have also shown that BACH1 inhibits angiogenesis. HIF1 induces the expression of *BACH1*, which then represses *HMOX1* in human endothelial cells (113). Because HO-1 promotes angiogenesis in human microvascular endothelial cells (114), BACH1 is expected to inhibit angiogenesis in these cells. In addition to HO-1, BACH1 also represses the expression of vascular endothelial growth factor gene *VEGFA* and interleukin *CXCL8* (IL-8), which are inducers of angiogenesis (40, 115, 116), and the angiopoietin-1 gene *ANGPT1* in pericytes (117). Angiogenesis in response to ischemia is also increased in *Bach1*^−/−^ mice compared with WT mice (118). However, whether or not these studies showing the inhibitory role of BACH1 on angiogenesis simply reflect its function in endothelial cells is unclear. Further studies will be needed to determine if BACH1 promotes angiogenesis in cancer tissues, perhaps as an extension of the regulatory role played by BACH1 and/or BACH2 in developmental angiogenesis.

### Promotion of proliferation by BACH1

Considering that cellular senescence is a tumor suppressor mechanism, BACH1 may promote carcinogenesis and cancer growth by suppressing cellular senescence. This function may also be important for the proliferation of established cancer cells, as senescence is induced in cancer cells in response to various stresses, including therapeutic interventions (119). BACH1 inhibits Ras^G12V^-induced cellular senescence (38) and enhances the tumor growth activity of primary mouse embryonic fibroblasts (120). The tumor suppressor protein p19ARF inhibits cell proliferation by blocking the binding of p53 to MDM2, the p53 ubiquitin E3 ligase, to stop degradation of p53 protein (121, 122). p19ARF also inhibits the binding of p53 to BACH1 (123). Therefore, increased activity of BACH1 in cancer cells may resist the p53 pathway and support proliferation of cancer cells under stressful conditions, such as exposure to ROS or anticancer therapeutics.

### Epigenetic stabilization of cellular properties by BACH1

The interaction of transcription factors with DNA is transient and highly dynamic (124). Therefore, changes in cancer cell properties instilled by nongenetic alterations likely involve mechanisms that transduce the transient interaction of transcription factors with DNA to more stable biochemical alterations. One such mechanism is the modification of histone and DNA, wherein BACH1 is involved through interacting with cofactors. Studies in development and embryonic stem (ES) cells suggest an epigenetic function of BACH1. BACH1 recruits polycomb complex 2 to promote H3K27me3 modification in ES cells and thus restrict mesendoderm differentiation (125). This function of BACH1 is also repurposed in cancer cells. Investigations in colorectal cancer and melanoma have shown that the heterodimer of BACH1 and MAFG recruits DNA methyltransferase DNMT3b to silence genes, including tumor suppressor genes (126, 127). Similar epigenetic regulation may also operate in other types of cancer. Especially, it will be important to investigate whether the repression of epithelial genes by BACH1 in cancer cells also involve epigenetic rewriting of the target genes. In this vein, HDAC1 is known to promote silencing of *CDH1* by SNAI1 as its corepressor (128, 129) and metastasis of PDAC (130) and liver cancer (131). Because HDAC1 is a corepressor of BACH1 (38), these observations suggest that the epigenetic regulators interacting with BACH1 may be involved in the pathogenic functions, including the promotion of EMT and metastasis.

### Putative tumor-suppressing roles of BACH1

In contrast to the cancer-promoting functions of BACH1 described above, its tumor-suppressing role has also been reported. A minor SNP allele of C (rs372883T>C) located in the 3′ UTR of the *BACH1* gene, which increases the BACH1 expression compared with the major T allele, is associated with a reduced risk of PDAC (132). BACH1 may also inhibit tumor initiation and cancer cell proliferation at an earlier stage.

In addition, BACH1 may impose negative effects on the cancer cell survival by affecting protein homeostasis. Cancer cells often become more dependent on the ubiquitin–proteasome system than normal cells (133, 134). This may be due in part to the production of unbalanced amounts of proteins in a particular protein complex or pathway or the production of abnormally high amounts of proteins, such as immunoglobulin in multiple myeloma cells. This increased dependency on ubiquitin–proteasome has been adopted for therapeutics in multiple myeloma and is now being clinically tested for prostate cancer, neuroendocrine tumors, and several types of lymphoma (135). Interestingly, the expression of proteasome components is generally increased in cancer cells. NRF1 (NFE2L1) and NRF3 (NFE2L3) activate the expression of these genes (136, 137, 138, 139, 140, 141, 142, 143) by directly binding to these genes. In contrast, the increased expression of proteasome genes in gallbladder cancer is due to a reduction in BACH1 activity rather than an increased activity of NRF1 (144). BACH1 directly represses the expression of a subset of proteasome genes in gallbladder cancer and cholangiocarcinoma cells (144, 145). Therefore, cancer cells, which express BACH1 at elevated levels, may be more sensitive to alterations in protein homeostasis and proteasome inhibitors because of the reduced expression of proteasome subunit genes. Whether or not BACH1 regulates the expression of proteasome genes in normal cells is unclear.

## New challenges for understanding BACH1 at crossroads of cancer biology

Functions of transcription factors often depend on cellular and environmental contexts, which reflect complex and dynamic networks generated by transcription factors with their regulators and target genes. Networks of BACH1 in cancer and normal cells appear to provide new research directions in cancer, which are discussed below with several hypotheses on the function of BACH1 in cancers. To frame these issues in previous findings, we propose a “two-faced BACH1 model” in cancer.

### The two-faced BACH1 model

**Figure 4:**
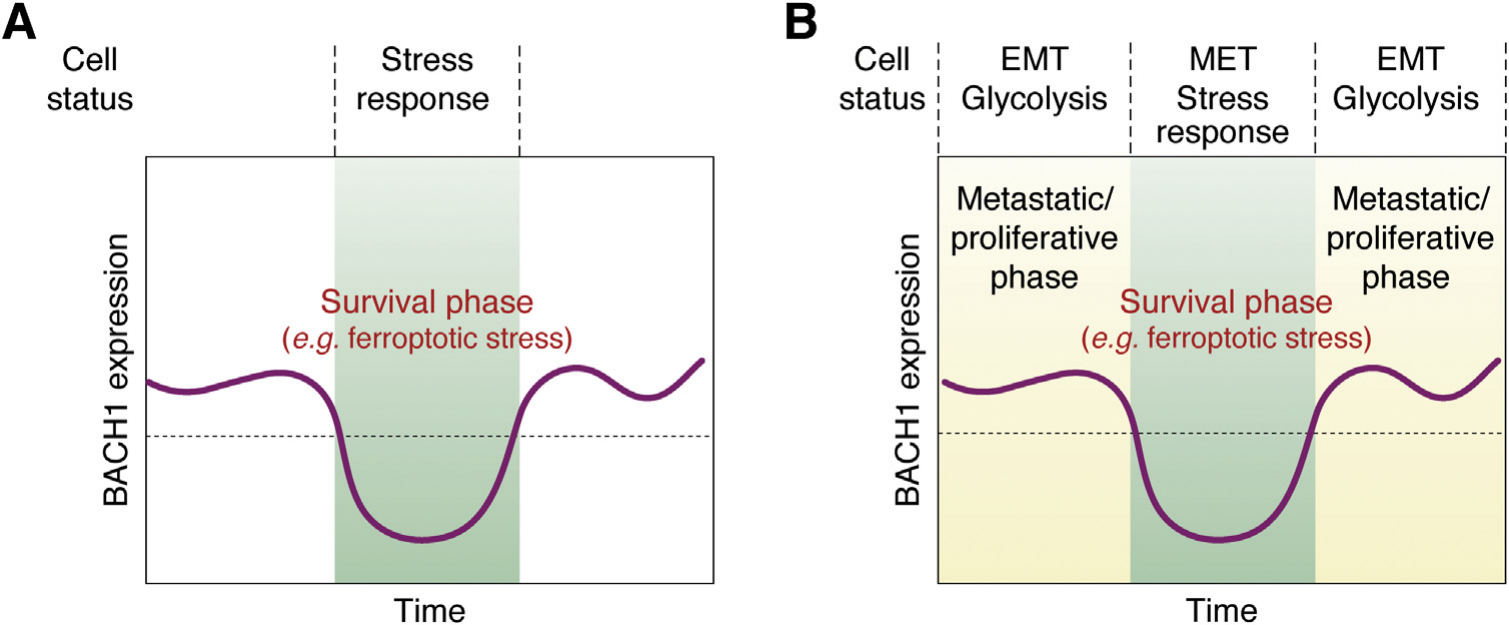
**The two-faced BACH1 model: BACH1 coordinates metastasis, proliferation, and stress response of cancer cells.***A*, a classical model of BACH1 explains that cytoprotective genes are induced when its activity or amount is reduced. *B*, a “two-faced BACH1 model” explains that BACH1 promotes proliferation and metastasis while repressing the expression of genes for cell survival against stressors. A reduction in its amount or activity leads to a stress response and survival of cancer cells, along with alterations in the cancer cell properties. This model demonstrates that BACH1 coordinates dynamic alterations of cancer cell phenotypes in response to oxidative, ferroptotic, and other stresses. BACH1, BTB and CNC homology 1.

One of the most important points is that BACH1 may function as a regulator modulating the cell fate of cancer cells and an inducer of cancer cell plasticity (Fig. 4). In the context of oxidative cell response, the reduction of BACH1 enables the induction of genes for cell protection, including *HMOX1, FTH1*, *FTL*, and *SLC40A1* (Fig. 4*A*). In this classical model, restricting the expression of the cell-protective genes under the normal conditions should reduce the burden of stress responses and thus indirectly allow utilization of resources, including metabolites, for cell proliferation and cell-specific functions. In addition to such a gate-keeper function of oxidative stress response, those studies discussed in this review have established that BACH1 (*i.e.*, its presence) further increases, directly and indirectly *via* the GRN, the expression of genes critical for cancer progression (Fig. 4*B*, a“two-faced BACH1 model”). When active, BACH1 not only induces the expression of genes for cancer progression but also represses the expression of a cohort of stress-responsive genes. Therefore, BACH1 is two-faced in that it promotes cancer progression at the expense of stress resilience. When BACH1-mediated regulation is weakened, stress response ensues for survival along with silencing of metastatic and other properties. Cancer cells are expected to exploit dynamic changes in gene expression caused by the flexible amount or activity of BACH1, which can be altered in response to oxidative and other stresses, for their progression and survival.

### Does BACH1 contribute to the dynamics and plasticity of EMT?

EMT is not a binary state of either epidermal or fully differentiated mesodermal cells but is composed of multiple transitionary states, which show different activities in tumor initiation and metastasis in animal models (105, 146, 147, 148, 149). While the very early stages of transition show metastatic activity, fully transitioned mesenchymal cells show less metastasis (105). ZEB1, ZEB2, SNAI1, and TWIST1 drive EMT of cancer cells, but they are not required for metastasis in animal models (150, 151). While metastasis of lung cancer in mouse models did not require ZEB1 and ZEB2, the metastatic cells nonetheless showed an altered gene expression reflecting, at least in part, EMT (150). Once metastatic cancer cells find a favorable niche, mesenchymal-to-epithelial transition (MET), the reverse process of EMT, is supposed to take place to resume proliferation there (5). Therefore, metastasis appears to involve dynamic and reversible changes in cell properties driven by multiple regulators that alter the gene expression. Considering the requirement for BACH1 in metastasis of various cancers, BACH1 may be one of factors that establishes the early stages of EMT in cancer cells.

The BACH1 GRN may also contribute to proceeding or backtracking through the steps of EMT based on its network structure. By functioning as both the activator and repressor, BACH1 can form a coherent feedforward loop for the regulation of EMT (Fig. 3*C*) (152). Because EMT requires both sufficient BACH1 and SNAI2 for the repression of epithelial genes, such a GRN precludes spontaneous EMT in cancer cells: EMT ensues only when enough BACH1 and SANI2 have accumulated within cells. In contrast, once a signal that increases BACH1 or SNAI2 is diminished, MET is expected to take place rapidly. A recent report described a phenomenon in which a fraction of clonal cancer cells undergoes changes in the gene expression that are dependent on a set of transcription factors and last beyond a single cell generation, causing cancer heterogeneity (153). Therefore, the BACH1 GRN may be a critical mechanism involved in the dynamic transition of EMT and MET in cancer cells. Understanding the regulation of EMT and MET is an important issue toward the heterogeneity of cancer cells within a tumor, as only a subpopulation of cancer cells in the tumor undergoes EMT.

The above considerations highlight the issue of how the expression and activity of BACH1 are regulated in the cancer microenvironment. In this regard, transforming growth factor-β, a critical growth factor regulating the EMT process (154) and cancer niche signaling (155), is interesting, as it induces the expression of BACH1 and MAFK (156). Another candidate may be the interferon response because BACH1 is induced by interferon β (157). Hypoxia, which is known to induce BACH1 expression (158), may also contribute to the regulation of BACH1. BACH1 may trigger switching between EMT and MET in response to changes in such environmental factors.

### Does BACH1 impose vulnerability of ferroptosis to metastatic cancer cells?

Ferroptosis, a necrosis-like type of programmed cell deaths (159), is attracting attention as a key mechanism involved in the development of future cancer therapies (160, 161, 162, 163). In ferroptotic cells, the accumulation of lipid peroxides and lipid hydroxyl radicals generated by iron leads to cell death (159, 164). Ferroptosis as well as apoptosis have been frequently shown to act as cancer suppressor mechanisms *in vitro* and *in vivo* (165, 166, 167, 168, 169, 170, 171, 172). Glutathione peroxidase 4 opposes ferroptosis by reducing lipid peroxides using GSH (166). BACH1 promotes ferroptosis by repressing the expression of genes involved in GSH synthesis and iron handling (173) (Fig. 3*D*). BACH1 represses the transcription of *SLC7A11* encoding the subunit of the transporter of cystine (System Xc-), which is used for GSH synthesis, and the modifier and catalytic subunits of glutamate–cysteine ligase (*GCLM* and *GCLC*), the rate-limiting enzyme of the GSH synthesis pathway (173, 174). While ferritin and ferroportin antagonize ferroptosis by reducing intracellular labile iron (163, 175), BACH1 represses the transcription of their genes (*FTH1*, *FTL*, and *SLC40A1*) (58, 59, 173, 174). Thus, BACH1 promotes ferroptosis by transcriptionally repressing cohorts of genes in the GSH synthesis pathway and labile iron metabolism. BACH1 further regulates another pathway for ferroptosis. Intercellular contacts mediated by E-cadherin suppress ferroptosis by activating the Hippo signaling pathway, leading to a reduced activity of YAP, which is a ferroptosis-inducing transcription coregulator. Upon the initiation of EMT, YAP is activated, leading to increased sensitivity toward ferroptosis (176). Because BACH1 inhibits the E-cadherin expression and intercellular adhesion (79) (Fig. 3*A*), it is reasonable to assume that BACH1 also inhibits the E-cadherin-Hippo-YAP pathway in addition to the cystine-GSH-glutathione peroxidase 4 pathway, thereby promoting ferroptosis. Conversely, under the induction of ferroptosis, BACH1 is degraded and the transcription of these genes is induced, causing cells to resist ferroptosis (173). In conclusion, BACH1 works as a potent promoter of ferroptosis by inhibiting all of the major cellular barrier mechanisms against ferroptosis (Fig. 3*D*).

The promotion of ferroptosis by BACH1 *via* multiple pathways may provide a therapeutic strategy for cancers. Cancer cells with a high expression of BACH1 may be more sensitive to ferroptosis-inducing drugs than those with low BACH1 levels. If we can inhibit the degradation of BACH1 in cancer cells upon induction of ferroptosis, the efficacy of anticancer therapies involving ferroptosis, not only cytotoxic drugs but also immune checkpoint therapies (177), will be further enhanced. Importantly, cancer cells that undergo EMT and have acquired resistance against molecular-targeted anticancer therapy become more susceptible to ferroptosis (178, 179, 180), suggesting ferroptosis induction as an attractive means of overcoming resistance to molecular-targeted therapies, including tyrosine kinase inhibitors. Given that BACH1 promotes both EMT and ferroptosis (79, 173), it is tempting to speculate that BACH1 is also involved in the increased susceptibility to ferroptosis after EMT. This hypothesis is further supported by the recent finding that a reduced expression of ferritin is a shared feature of the EMT transcriptional response in diverse cancer cells (106). BACH1 may bring both EMT and ferroptosis sensitivity to metastatic cancer cells. This line of research will also unveil the long-standing conundrum of why cancer cells tend to accumulate iron (161).

### Is BACH1 involved in cancer cell stemness?

EMT also drives cancer stemness and resistance to therapies (7, 146, 150, 151, 181, 182, 183). SNAI2 and TWIST1 induce the stemness property of cancer cells (184, 185). Interestingly, BACH1 stabilizes the pluripotency factors NANOG, SOX2, and OCT4 in human ES cells by interacting with them and recruiting deubiquitinase USP7 for deubiquitination of these factors (125). Therefore, BACH1 may also promote the expression of stem cell–like properties in cancer cells by stabilizing pluripotency transcription factors. This possibility is interesting, as it can explain the regulatory connection between EMT and stem cell properties.

### How does BACH1 activate target genes?

How does BACH1 transactivate some of its target genes? BACH1 collaborates with HOXB8 in metastasis of colorectal cancer. The *HOXB8* gene is required to maintain the malignant features of colorectal cancer cells, including invasion and migration *in vitro* and tumor formation and metastasis in a mouse xenograft model (186). HOXB8 directly activates the expression of *BACH1*. The reduced cell mobility upon HOXB8 knockdown is rescued by BACH1 expression. These observations suggest that *BACH1* is a critical downstream target gene of HOXB8 in metastasis. Furthermore, HOXB8 and BACH1 interact with each other and bind together to many of the HOXB8 target genes, including *BACH1* itself (186). BACH1 may cooperate with HOXB8 to induce the expression of their shared target genes. BACH1 interacts directly with the MLL complex that catalyzes the trimethylation of histone H3 lysine 4 and is required for the expression of a set of BACH1 target genes (36). Cancer cells may utilize such an interaction to convert BACH1 into an activator.

It is important to compare absolute numbers of protein molecules in cancer cells. In hematopoietic cells and erythroid cells, BACH1 and its competing factor NF-E2 p45 are present around 500 and 10,000 molecules per nuclei, respectively. In contrast, their coregulators show opposite trends: repressive cofactor HDAC1, >63,000 molecules and activating cofactor histone acetyltransferase p300, 7000 molecules (187). Therefore, nuclei are full of repressive cofactors and have a shortage of activating cofactors. This may facilitate the gene repression by BACH1, although it is present in much lower absolute protein numbers than NF-E2 p45 in hematopoietic cells. At the same time, a slight decrease in the BACH1 content may shift from repression to activation of its target genes. To understand how BACH1 shuts off its interactions with corepressors to transactivate gene expression, we need first to quantify those protein molecules in cancer cells.

### Does the function of BACH1 in mitosis contribute to cancer progression?

BACH1 has functions beyond transcriptional regulation. BACH1 contributes to the stability of the cell division axis through its interaction with the hyaluronan-mediated motility receptor (HMMR) (188, 189). This may be part of the mechanism by which BACH1 supports cell proliferation. This function of BACH1 requires the phosphorylation of the region of BACH1 between the bZip and CLS domains (C-terminal side of BACH1, Fig. 2*A*). Suppression of the C-terminal phosphorylation instead results in stronger binding to MAFK (189). This suggests that BACH1 switches its functions between a transcription factor and a mitotic axis regulator *via* the phosphorylation and dephosphorylation of its C-terminal side. The effect of this transcription-independent function of BACH1 on cancer biology has not yet been reported. HMMR is involved in the oscillation of mitotic chromosomes, which suppresses chromosome missegregation (190, 191). Therefore, BACH1 may suppress mitotic catastrophe with HMMR. Changes in this mitotic function of BACH1 may facilitate oncogenesis by inducing improper segregation of chromosomes. The multifaceted roles of BACH1 in cancer biology should be further explored.

## Concluding remarks

The recent research summarized here has established the critical roles of BACH1 in cancer. The two-faced BACH1 model posits that both the increase and the decrease of BACH1 activity are involved in the progression of cancers but with distinct expression of cancer cell properties. Thus, the presumed differences in the activity of BACH1 may confer unique vulnerabilities to cancer cells. For example, when one is to target BACH1 as a treatment to inhibit metastasis, a combination of drugs that suppress stress responses may bring more effective therapeutic effects. Further understanding of the BACH1 GRN in cancer cells will allow a rational development of therapeutic strategies. More generally, two lines of research on transcription factors are needed.

The concept of cellular plasticity has expanded from referring to just embryonic cells to include any cells in the body and covers the dedifferentiation, transdifferentiation, and phenotypic transition of differentiated cells (*e.g.*, EMT). Fundamental studies have shown that some transcription factors can determine cell fate and that the cell fate is much more plastic than previously considered. While regulation by transcription factors is often regarded as secondary to that by chromatin, the truth is that transcription factors direct chromatin accessibility in the context of development and differentiation (192). Only four transcription factors induce pluripotent stem cell reprogramming (193). Given the role of transcription factors as master regulators of cell fate, their pharmacological modulation has been widely studied, including the inhibition of DNA binding or protein interaction using small molecules (194). To increase the activity, transcription factors themselves can be used as drugs, in either their protein form or encoded in suitable gene vectors (195). These successful efforts have debunked the previous view that transcriptional factors are undruggable.

The human genome encodes 1639 known or likely transcription factors (21), but research in cancer biology is heavily skewed toward a small subset of transcription factors whose importance in cancer has been well established. As many of these transcription factors likely contribute to the homeostasis and/or stress responses of normal and cancer cells, further understanding of their functions will be crucial. As our understanding of human transcription factors and their GRNs expands, we will be able to understand how cancer cells become so deadly and how we can avoid the dreadful fate they impose.

## Conflict of interest

The authors declare that they have no conflicts of interest with the contents of this article.
